# Treatment of Resistant Depression: A Pilot Study Assessing the Efficacy of a tDCS-Mindfulness Program Compared With a tDCS-Relaxation Program

**DOI:** 10.3389/fpsyt.2019.00730

**Published:** 2019-10-23

**Authors:** Aurore Monnart, Marie-Anne Vanderhasselt, Elisa Schroder, Salvatore Campanella, Philippe Fontaine, Charles Kornreich

**Affiliations:** ^1^ Laboratory of Psychological Medicine and Addictology, CHU Brugmann - ULB Neuroscience Institue (UNI), University of Brussels (ULB), Brussels, Belgium; ^2^ Department of Head and Skin, Ghent Experimental Psychiatry Lab, Ghent University (UGent), Ghent, Belgium; ^3^ Department of Experimental Clinical and Health Psychology, Ghent University, Ghent, Belgium; ^4^ Department of Psychiatry, CHU de Charleroi, Charleroi, Belgium

**Keywords:** major depressive disorder, transcranial direct current stimulation (tDCS), mindfulness-based cognitive therapy, rumination, cognitive control

## Abstract

**Background:** This pilot study explores a therapeutic setting combining transcranial direct current stimulation (tDCS) and mindfulness-based cognitive therapy (MBCT) for patients with drug-resistant depression. tDCS has shown efficacy for depression treatment and improvement could be maintained with the combination with mindfulness, which has shown depression relapse-prevention properties.

**Methods:** Thirty-one treatment-resistant depressed patients have been assigned to our experimental treatment condition [tDCS combined with MBCT (*n* = 15)] or to a control condition [tDCS combined with relaxation (*n* = 16)]. Patients have completed both an intensive treatment block (eight consecutive days) and a single remind session 2 weeks after the intensive treatment. Clinical (depression, anxiety, and rumination) and cognitive (general cognitive functioning, mental flexibility, and working memory) symptoms of depression have been assessed through different questionnaires at baseline (t0), after the first block of treatment (t1), and after the remind session (t2).

**Results:** Results seem to indicate a positive impact of both treatment conditions on clinical and cognitive symptoms of depression at t1. However, the treatment condition combining tDCS with mindfulness has been found to better maintain clinical improvements at t2 regarding some clinical [Montgomery–Åsberg Depression Rating Scale (MADRS) and Sadness and Anger Ruminative Inventory (SARI)] and cognitive variables (Digit Span-F and Digit Span-B).

**Conclusion:** Based on the current observations, a multi-disciplinary treatment approach combining tDCS and MBCT might be effective in resistant depressed patients in the long run, even though further clinical research is necessary.

## Introduction

Major depressive disorder (MDD) is a highly prevalent condition in which patients experience a daily sustained negative affect and a persistent reduction in positive affect. Diverse questions remain to be elucidated to find out why those depressed individuals cannot just swap out of negative vibes. However, until now, MDD is mostly considered as a disease of impaired emotion regulation characterized by excessive negative ruminations hijacking the patients’ mental life and impacting their mood. As a matter of fact, rumination is now regarded as a maladaptive emotion-regulation strategy at the center of depression processes (1), which mainly increases the hallmark symptoms of depression (for a meta-analysis, see 2).

### Ruminations: Cognitive Deficits and Modified Brain Activity

Various alterations in cognitive functioning indexed by modified brain activity are thought to subtend the use of this maladaptive emotion-regulation strategy instead of other adaptive ones. Indeed, some specific cognitive deficits have been identified as potentially leading people to engage in ruminative processes (3). For instance, it has been shown that deficits in attention and working memory make negative content more accessible to depressed individuals, as negative mood has been found to be more frequently related to negative attention bias towards emotional information (4, 5) and to greater accessibility of negative memories (6; for a review, see 7). Last but not least, deficits in cognitive control and, particularly, cognitive inhibition (one of its pivots) have been identified in patients with MDD and are thought as a potentially main causal factor in rumination (8–11; cited in 12). Importantly, rumination and linked cognitive deficits have been related to a particularly modified brain activity: a hyper-activated amygdala region, which is insufficiently controlled by an hypo-activated prefrontal region (13), leading to an unsatisfactory cognitive control of emotions (3–11, 12, 14).

### Treatments: Medication, Psychotherapy, and Neuromodulation Techniques

Those observations are probably relevant enough to impact depression treatments. As a matter of fact, if usual treatments for the disease directly target the limbic hyperactivity through pharmacotherapy (i.e., antidepressant medication), the common use of antidepressant medication, sometimes combined with psychotherapy, leaves numerous patients with unsatisfactory responses in terms of both remission and relapse and chronicity (15). Some researchers even consider that two of three MDD patients retain with depression symptoms after a first-line treatment (16, 17). Moreover, depression often shows a chronic course and various relapses (5, 18). Apart from their insufficient efficacy, antidepressant medications are often characterized by important secondary effects (19, 20) leading to a weak treatment adherence (21). Therefore, noninvasive brain stimulation techniques (NIBS), such as repetitive transcranial magnetic stimulation (rTMS) and transcranial direct current stimulation (tDCS), have been raising interest for their potential therapeutic efficacy in different psychiatric and neurological conditions. Indeed, in many neuropsychiatric diseases, such as depression, an important pathophysiological factor has been identified: the alteration of neuroplasticity and of cortical excitability. About 50 years ago, some researchers had already observed that the application of transcranial direct current can induce a sufficiently important current flow to achieve physiological and functional effects in both healthy subjects and patients with psychiatric disorders (22). While this technique (tDCS) has been almost forgotten for the following years, more recent studies have redefined it as a tool to modulate human brain activity, and its physiological effects are starting to be systematically explored in various researches (22). In depressed patients, tDCS has especially been used in order to prime the neural system and to reinforce its hypo-activated prefrontal executive functioning (22). As a matter of fact, the hypo-activated prefrontal region (13), by insufficiently controlling the hyper-activated amygdala region, has been related to ruminations, which is considered as a main causal and maintenance factor of depression.

### About tDCS in the Treatment of Depression

tDCS works by inducing neuronal plasticity *via* application of relatively weak currents through the scalp. It uses a constant low current (1–2 mA) delivered directly to the brain area of interest *via* electrodes positioned on the scalp, inducing intracerebral current flows (23). The device has an anodal electrode (the positively charged electrode, placed over the region of interest), which increases the excitability of the underlying cortex (24), and a cathodal electrode (the negatively charged electrode, placed in another location in order to create a circuit), which decreases the excitability of the underlying cortex (24).

Historically, Fregni and collaborators (25) were the first to describe the antidepressant effects of anodal tDCS over the right prefrontal dorsolateral cortex (rPFDLC). Since then, encouraging but controversial results have been found regarding the clinical outcomes of the application of tDCS in patients suffering from MDD. Indeed, different meta-analyses have been published. A recent one concluded to a probable efficacy of anodal tDCS of the left dorsolateral prefrontal cortex (DLPFC) (with right orbitofrontal cathode) in major depressive episode without drug resistance but no clinical effects of the anodal tDCS of the left DLPFC in drug-resistant major depressive episodes (22). Another recent meta-analysis concluded at a moderate efficacy of active tDCS in MDD compared with placebo stimulation (sham stimulation: inactive tDCS; in this kind of placebo stimulation, a very short duration current is applied that produces a sensation for the patients, but this current stops really quickly) (26). In fact, studies have explored the therapeutic effects of tDCS in different depressed patient samples (e.g., non-drug-resistant depression vs drug-resistant depression, and unipolar vs bipolar depression) in order to compare its efficacy to pharmacotherapy and/or as an add-on treatment to antidepressant medication (22). tDCS has showed antidepressant efficacy when used alone (27), but a significant synergic effect of active tDCS combined with classic antidepressant medication has been found in different studies (28). For instance, in 2012, Brunoni et al. (29) have showed that a combined use of antidepressants and active tDCS offers better results than each modality alone. In 2013, the same research group has found a greater mood improvement after active tDCS combined with sertraline compared with all other groups. Importantly, the same study showed that active tDCS was significantly superior to placebo, but no differences were found between active tDCS and sertraline conditions (30). Besides, one of the most important studies to date was led on a sample of 245 depressed patients who were assigned to different experimental conditions [high level of escitalopram (20 mg); high level of active tDCS – 1 stimulation per day for 15 days; 30 min; 2 mA; antidepressant placebo; sham stimulation with tDCS]. This study concluded to a higher efficacy of active tDCS compared with placebo condition but to a lower efficacy compared with escitalopram (20 mg). According to those results, active tDCS should then not be as effective as classic antidepressant medication (31). Importantly, some researchers have proposed that tDCS efficacy seems to diminish in patients with high therapeutic resistance (32). It remains to be clarified whether the probable therapeutic effects of tDCS are clinically meaningful and how to optimally perform tDCS in therapeutic settings for drug-resistant patients.

Significantly enough, to date, only few studies have explored a possible synergic effect between tDCSs and psychotherapeutic modalities in the treatment of depression. Moreover, researches exploring the combination between tDCS and various forms of cognitive therapies displayed contradictory results (26, 33).

### About Mindfulness-Based Cognitive Therapy in the Treatment of Depression

This last decade, a specific version of cognitive therapy, mindfulness-based cognitive therapy (MBCT) (34), has been arousing interest in treating depression by preventing relapses (18, 35–41). MBCT, defined as the non-elaborative and non-judgmental awareness that arises from deliberately paying attention to the present-moment experience with the attitude of curiosity and acceptance (42), has been associated with depression relapse-prevention properties by targeting the prefrontal executive functioning.

Indeed, the MBCT program developed by Segal et al. (34) consists of a group therapy of eight sessions (one session per week) in which the objective is for patients to learn to reckon and identify their cognitive and behavioral reactions following a low mood moment and/or in stressful situations and to consider them with an attitude of acceptance and kindness. This technique actually teaches MDD patients to disengage from negative automatic ruminations by training their attention and executive functioning abilities (34, 43–46). MBCT has also been shown to improve working memory abilities and to lead to a cerebral plasticity process by targeting the DLPFC and leading to higher activation of the DLPFC in some error-detection tasks after completing an MBCT program and an increase of the fronto-insular cerebral circuits when treating emotional negative stimuli. Those cerebral changes following MBCT program have been shown to be directly proportional to the amount of mindfulness practice (47).

It appears that tDCS has showed some efficacy as an antidepressant treatment (but the questions about its sustained effect through time remain unanswered) and that MBCT has been shown to be effective in preventing relapses, and we wanted to test their combined use. Furthermore, we suggest that we could optimize MBCT sessions in depressed patients if their hypo-activated neural system of PFDLC has first been primed with tDCS. That is, we think that tDCS application on patients’ PFDLC should optimize the ability to perform the MBCT program by allowing the patients to improve their attentional, cognitive inhibition and executive functioning abilities. To our knowledge, MBCT use in combination with tDCS has never been studied. Relaxation has been considered in our study as a placebo condition for MBCT. Indeed, somatic relaxation is a primarily body-awareness-based relaxation intervention (48). It consists in progressive muscle relaxation (using tensions and release of muscles throughout the body to relax), simple breathing techniques, and guided imagery to give a comprehensive course on stress reduction (i.e., focus on bodily relaxation). It has previously been used as a sham condition in studies on mindfulness effects (49, 50). If tDCS and MBCT act complementarily by both influencing the same neural circuits, we would expect an improvement of cognitive deficits in depressed patients, a diminution of ruminations, and lower depression scores after tDCS treatment to be better maintained through time in the MBCT condition compared with the relaxation condition. Indeed, we suggest that tDCS application on the left PFDLC of resistant depressed patients should optimize the ability to perform the MBCT program by allowing the patients to improve better than during the relaxation sessions, their attentional, cognitive inhibition and executive functioning abilities and by allowing them to disengage from automatic negative ruminations, which is maintaining their depression.

## Method

### Trial Design and Randomization

Treatment-resistant MDD patients were recruited by qualified psychiatrists on a clinical basis through the psychiatric services of CHU Brugmann (Brussels, Belgium) and Hospital Vincent Van Gogh (Marchienne-au-Pont, Belgium) from June 2015 to December 2016, as part of an initial pilot study. After a screening procedure led by the investigator (a clinical psychologist), selected patients were then successively integrated in different treatment groups following their inclusion order in the protocol. Each group was designed to include six patients. A total of eight treatment groups were necessary to include the initially recruited 46 patients. Recruited patients have been randomly assigned to two experimental conditions: (1) the first group has received active tDCS on the left DLPFC (20 min; 2 mA) directly followed by 2-h MBCT group sessions; (2) the second group has received active tDCS on the left DLPFC (20 min; 2 mA) directly followed by a 30-min Jacobson relaxation session (**Figure 2**, randomization protocol). Due to the protocol structure and clinical implications, it was impossible for the investigator to be blinded regarded the groups of enrolment (relaxation or mindfulness). Patients were informed during the first session that they were going to undergo relaxation or mindfulness. There were no important changes to methods after pilot trial commencement.

### Participants

Forty-six treatment-resistant MDD patients have been recruited by qualified psychiatrists on a clinical basis through the psychiatric services of CHU Brugmann (Brussels, Belgium) and Hospital Vincent Van Gogh (Marchienne-au-Pont, Belgium). A screening procedure led by the investigator confirmed the inclusion criteria for all participants. That is, patients matched the following criteria: (1) diagnosis of non-psychotic unipolar MDD according to the *Diagnostic and Statistical Manual of Mental Disorders*, 5th Edition (DSM-V), confirmed by the Mini-International Neuropsychiatric Interview (MINI) and with a minimal depression baseline score of 15, which has been defined as the minimal score for the threshold of depression, at the Montgomery–Åsberg Depression Rating Scale (MADRS); (2) comorbidities with anxiety disorders and personality disorders, according to the DSM-V were allowed, but patients with other comorbidities, such as psychosis and addiction diseases, were excluded; (3) patients with any actual or antecedent of severe somatic and/or neurological diseases were excluded; (4) resistant depression has been defined as a failure to respond to at least two antidepressant medications; and (5) patients included in our study have been allowed to continue their personal antidepressant medication, which has been controlled to be stable for at least 6 weeks before beginning and during the treatment. Benzodiazepines have been accepted up to a maximal dose of 20 mg diazepam equivalent per day. (6) Patients have been allowed to pursue their eventual individual psychotherapy, except for cognitive-behavioral therapy (CBT); and (7) patients with a previous background of mindfulness and/or neurostimulation techniques have been excluded. Note that the local ethics committees of CHU Brugmann and Hospital Vincent Van Gogh approved the study. Informed written consent to participate in the study was obtained from all participants after they received all aspects regarding the procedure details and the aims of the study. As mentioned before, among those 46 patients, 15 dropped out of our research protocol mostly due to their work schedules and to the highly energy-demanding protocol, so 31 patients were finally included in the present experiment.

### Procedures

Our main aim was to assess the impact of “combining MBCT with active tDCS.” The efficacy of this combined treatment (active tDCS on the DLPFC + MBCT) on the cognitive and clinical symptoms of depression was then investigated by comparing it with a procedure in which active tDCS was coupled with a “non-MBCT technique,” that is, relaxation (which was classically used as a sham condition in studies on mindfulness effects; 49, 50). Therefore, 31 patients have been randomly assigned to two experimental conditions: (1) the first group has received active tDCS on the left DLPFC (20 min; 2 mA) directly followed by 2-h MBCT group sessions (MBCT condition 1; *n* = 15), and (2) the second group has received active tDCS on the left DLPFC (20 min; 2 mA) directly followed by a 30-min Jacobson relaxation session (relaxation condition; *n* = 16).

### Interventions

Patients from the two experimental conditions have been invited to take two blocks of treatment. That is, in block 1, patients have been invited to eight consecutive days where they have received daily an individual active tDCS on the left PFDLC (20 min; 2 mA) directly followed by a 2-h MBCT group session (daily session inspired by the program developed by 34) or a 30-min relaxation session (51; and in block 2, patients received a remind session 2 weeks after the first block of treatment.

For the tDCS, participants were seated in a circle in a group including between three and six people in a resting room with small lighting. Each participant received daily a 2-mA tDCS for 20 min (i.e., 1,200 s), with a fade-in period of 15 s and a fade-out period of 15 s, during eight consecutive open days. The anode was systematically placed over the left PFDLC and the cathode over the right PFDLC anatomically located by the use of a Quik-Cap referring to the electroencephalography international system 10–20 (anode located on F3 and cathode located on F4) (52). Both electrodes have been inserted in sponges soaked with saline solution to improve the conductivity and increase the patients’ comfort by limiting the risk of cutaneous lesion, before being placed over their respective brain areas. After having received tDCS, participants were invited either to a 2-h MBCT session or to a 30-min Jacobson relaxation session depending on the experimental condition they were assigned to. For each experimental condition (MBCT or relaxation), sessions were moderated by the same clinical psychologist and took place in the same resting room with small lighting and controlled temperature. Also, the same material was used for each experimental condition: a yoga mat and a cushion per participant. Note that if, according to the MBCT program developed by Segal et al. (34), the eight classical MBCT sessions are normally offered at the weekly pace, in our research protocol, those eight sessions have been offered at a daily rhythm right after the active tDCS, in order to comply with the usual tDCS protocols used in depression.

### Assessment and Outcomes

First, candidates for our study have been meeting for a 1-h appointment (screening) with a clinical psychologist in order to confirm the diagnosis of unipolar MDD according to DSM-V both through a psychological evaluation and through the MINI. Candidates matching the inclusion criteria were then selected for our research protocol. The day before the treatment beginning (t0), after receiving all study details and signing the informed consent form, every participant has been individually asked to complete both clinical and cognitive scales. First, they have been asked to complete the clinical questionnaires that aimed to assess their clinical state. Then, they have been asked to complete cognitive questionnaires in the presence of the same clinical psychologist.

**Figure 1:**
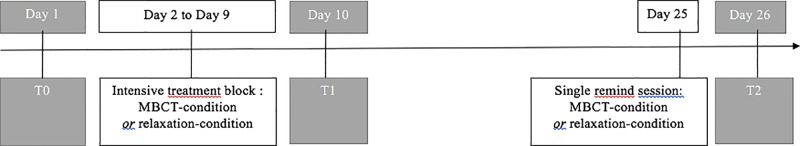
Treatment (white boxes) and assessment timeline (grey boxes). Treatment (white boxes) and assessment (grey boxes) timeline for both treatment conditions.

Clinical outcomes (depression, anxiety, and rumination) have been assessed through different clinical scales the day before the first block treatment (T0), the day right after the end of the first block treatment (T1), and the day right after the first remind session (T2) (**Figure 1**): MADRS (53), State-Trait Anxiety Inventory (STAI; 54), and the Sadness and Anger Ruminative Inventory (SARI; 55). Cognitive outcomes (general cognitive functioning, attention, working memory, and mental flexibility) have been assessed through different cognitive tests: Montreal Cognitive Assessment (MOCA; 56), Digit Span [Wechsler Adult Intelligence Scale (WAIS-IV); (57) and Trail Making Test (TMT; 58).

The primary efficacy outcome shall be assessed by the MADRS. The secondary outcomes will be assessed by the STAI and the SARI, and cognitive scores by MOCA, the Digit Span (WAIS-IV), and the TMT.

For the primary and secondary efficacy outcomes, obtained data in each experimental condition (group 1 and group 2) were analyzed with mixed, repeated-measures of variance model with scale scores as dependent within-subjects variables and treatment condition as between-subjects variable. Simple effects were inspected together with systematic examinations of interaction sources. Independent samples Student’s *t*-tests, chi-square tests, and Bonferroni’s *post-hoc* *t*-tests were used when relevant. All data analyses were performed with SPSS 20.00 at the level of significance set at 0.05.

### Analyses

Obtained data in each experimental condition (group 1 and group 2) were analyzed with mixed, repeated measures of variance model with scale scores as dependent within-subjects variables and treatment condition as a between-subjects variable. Simple effects were inspected together with systematic examinations of interaction sources. Independent samples Student’s *t*-tests, chi-square tests, and Bonferroni’s *post-hoc t*-tests were used when relevant. All data analyses were performed with SPSS 20.00 at the level of significance set at 0.05.

## Results

### Baseline Demographic, Clinical, and Cognitive Variables

Baseline demographic, clinical, and cognitive variables of the two groups (MBCT condition and relaxation condition) were analyzed by means of independent samples Student’s *t*-tests and chi-square tests when relevant. At baseline, MDD patients of the MBCT condition and the relaxation condition were similar in terms of demographic variables (age, gender, and educational level), of clinical variables (MADRS: hetero-evaluation of depression; STAI-E: auto-evaluation of state anxiety; SARI: auto-evaluation of sadness ruminative behavior), and of cognitive variables (MOCA: general cognitive functioning; TMT: mental flexibility; Digit Span: working memory), confirming the correct matching between groups. All patients’ demographic, clinical, and cognitive baseline scores and *p*-value, obtained through independent samples Student’s *t*-tests, and chi-square tests when relevant, are summarized in **Table 1**.

**Table 1:** Comparison of demographic, clinical, and cognitive baseline scores (t0) between participants of the two treatment conditions.

Variables	Treatments conditions	*N*	*M* (SD) or *N*	v: Degrees of Freedom	*t*-values	*p*-values
Demographic characteristics						
Age	MBCT condition	15	50 (5.38)	29	−0.16	0.901 > 0.05
	Relaxation condition	16	50,31 (8.08)			
Gender	MBCT condition	15	*N* = 5/10 (M/F)	1	0.059	0.809 > 0.05
	Relaxation condition	16	*N* = 6/10 (M/F)			
Educational level	MBCT condition	15	13.73 (2.73)	29	0.652	0.520 > 0.05
	Relaxation condition	16	13.06 (2.97)			
Clinical and antidepressant treatment characteristics						
SSRI	MBCT condition	15	*N* = 9	/	/	/
	Relaxation condition	16	*N* = 8			
SNRI	MBCT condition	15	*N* = 5	/	/	/
	Relaxation condition	16	*N* = 5			
SNRI + SSRI	MBCT condition	15	*N* = 1	/	/	/
	Relaxation condition	16	*N* = 2			
Comorbidities OCD	MBCT condition	15	*N* = 2	/	/	/
	Relaxation condition	16	*N* = 1			
Comorbidities with social phobia	MBCT condition	15	*N* = 0	/	/	/
	Relaxation condition	16	*N* = 1			
Clinical scores						
MADRS	MBCT condition	15	23.13 (6.8)	29	−0.641	0.526 > 0.05
	Relaxation condition	16	24.75 (7.20)			
STAI-E	MBCT condition	15	57.20 (9.15)	29	−0.983	0.334 > 0.05
	Relaxation condition	16	54.56 (6.61)			
STAI-T	MBCT condition	15	46.40 (9.88)	29	0.924	0.363 > 0.05
	Relaxation condition	16	51.06 (15.67)			
SARI	MBCT condition	15	40.53 (7.03)	29	−1.576	0.126 > 0.05
	Relaxation condition	16	45 (8.6)			
Cognitive scores						
MOCA	MBCT condition	15	26.87 (1.72)	29	1.599	0.121 > 0.05
	Relaxation condition	16	25.38 (3.2)			
TMT (B-A)	MBCT condition	15	45.67 (31.29)	29	−1.004	0.324 > 0.05
	Relaxation condition	16	59.88 (45.64)			
DS-F	MBCT condition	15	8.07 (1.9)	29	1.023	0.315 > 0.05
	Relaxation condition	16	7.44 (1.5)			
DS-B	MBCT condition	15	5.60 (1.95)	29	0.551	0.586 > 0.05
	Relaxation condition	16	5.25 (1.57)			

MBCT, mindfulness-based cognitive therapy; SSRI, selective serotonin reuptake inhibitor; SNRI, serotonin and norepinephrine reuptake inhibitor; OCD, obsessive compulsive disorder; MADRS, montgomery–åsberg depression rating scale; STAI, state-trait anxiety inventory; SARI, sadness and anger ruminative inventory; MOCA, montreal cognitive assessment; TMT, trail making test; DS-F, Digit Span Forward and DS-B, Digit Span Backward.

### Impact of the Two Treatment Conditions [MBCT Condition (*n* = 15) and Relaxation Condition (*n* = 16)] on Clinical and Cognitive Symptoms of Depression After T1 and T2

With the idea of assessing an eventual immediate (t1) and longer-term (t2) treatment conditions’ effect on clinical and cognitive symptoms of refractory depressed patients, we analyzed their clinical and cognitive scores with mixed, repeated measures of variance model with one dependent within-subjects variable [time (three levels: t0, t1, and t2)] and a between-subjects variable [treatment conditions (two groups)]. Obtained results are presented below both for the clinical assessment battery and for the cognitive assessment battery. Statistical corrections were applied when necessary. Means and SD scores of each clinical and cognitive scales at T0, T1 and T2 are presented for each treatment conditions in **Table 2**.

**Table 2:** Patient’s clinical and cognitive baseline scores (t0) and scores’ evolution at t1 and t2 for the two experimental conditions (MBCT condition and relaxation condition).

		T0		T1		T2	
Clinical variables		*N*	*M (SD)*	*N*	*M (SD)*	*N*	*M (SD)*
MADRS	MBCT condition	15	23.13 (6.8)	15	13.73 (9.57)	15	9.80 (9.93)
	Relaxation condition	16	24.75 (7.20)	16	17.25 (7.38)	16	19.06 (8.10)
STAI-T	MBCT condition	15	57.20 (9.15)	15	56.07 (11.12)	15	49.67 (11.08)
	Relaxation condition	16	54.56 (6.61)	16	52.63 (6.84)	16	53.31 (7.14)
STAI-E	MBCT condition	15	46.40 (9.88)	15	37.80 (10.95)	15	37.80 (9.37)
	Relaxation condition	16	51.06 (15.67)	16	43.81 (14.36)	16	45.56 (13.24)
SARI	MBCT condition	15	40.53 (7.03)	15	34.67 (10.16)	15	33.53 (10.82)
	Relaxation condition	16	45 (8.6)	16	40.50 (10.16)	16	43.69 (10.17)
Cognitive variables		*N*	*M (SD)*	*N*	*M (SD)*	*N*	*M (SD)*
MOCA	MBCT condition	15	26.87 (1.72)	15	28.87 (1.76)	15	29.60 (0.82)
	Relaxation condition	16	25.38 (3.2)	16	26.13 (2.87)	16	26.94 (2.46)
TMT (B-A)	MBCT condition	15	45.67 (31.29)	15	34.67 (28.47)	15	34.93 (21.76)
	Relaxation condition	16	59.88 (45.64)	16	42.25 (42.15)	16	40.63 (37.26)
DS-F	MBCT condition	15	8.07 (1.9)	15	9.4 (2.19)	15	10.33 (2.16)
	Relaxation condition	16	7.44 (1.5)	16	7.88 (1.74)	16	7.56 (2.06)
DS-B	MBCT condition	15	5.60 (1.95)	15	6.4 (2.23)	15	7.53 (2.13)
	Relaxation condition	16	5.25 (1.57)	16	5.38 (1.7)	16	5.38 (1.54)

MBCT, mindfulness-based cognitive therapy; MADRS, montgomery–åsberg depression rating scale; STAI, state-trait anxiety inventory; SARI, sadness and anger ruminative inventory; MOCA, montreal cognitive assessment; TMT, trail making test; DS, digit span.

#### Clinical Battery

A time effect was found between t0, t1, and t2 regarding all clinical scores in both treatment conditions, that is, STAI-E scores [*F*(2, 58) = 12.755; *p* < 0.001***; *η*2 = 0.305; *p* = 0.996], MADRS [*F*(1,496, 43,391) = 49.72; *p* = 0.001***; *η*2 = 0.632; *p* = 1.000], and SARI [*F*(1,976, 57,310) = 12.679; *p* = 0.001***; *η*2 = 0.304; *p* = 0.995].

No interaction effect between our within-subjects and between-subjects variables (time * treatment conditions) was observed for the STAI-E [*F*(2, 58) = 0.408; *p* = 0.640 > 0.05; *η*2 = 0.014; *p* = 0.113], while an interaction effect between our within-subjects and between-subjects variables (time * treatment conditions) has been found regarding the MADRS [*F*(1,496, 43,391) = 7.252; *p* = 0.002**; *η*2 = 0.200; *p* = 0.924] and the SARI [*F*(1,976, 57,310) = 3.709; *p* = 0.03*; *η*2 = 0.304; *p* = 0.995].

Interestingly, when looking at automatically generated pairwise comparisons for the clinical evolution at the STAI-E, we observe that this score is significantly different between t0 and t1 (*p* = 0.001***), not significantly different between t1 and t2 (*p* = 1.000 > 0.05), and significantly different between t0 and t2 (*p* = 0.005**), indicating a significant improvement of state anxiety at t1 and a stabilization of this score at t2 for both treatment conditions.

With the idea of exploring the observed interaction effect (time * treatment conditions) for the MADRS and SARI, we conducted specific post-hoc analyses. After splitting our general sample into two independent samples, we conducted an independent *t*-test.

**Figure 2:**
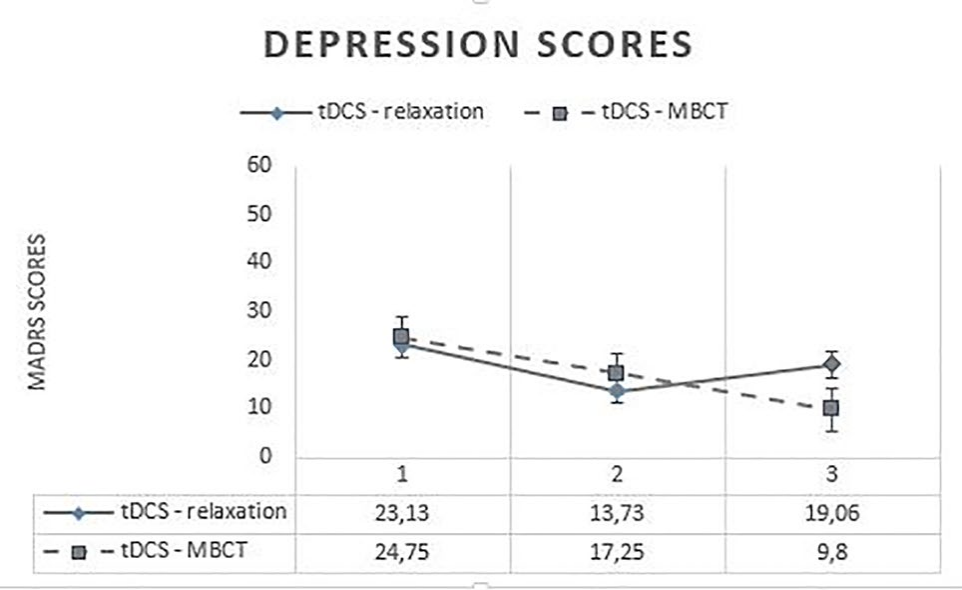
Evolution of depression scores across time. MADRS, Montgomery–Åsberg Depression Rating Scale; tDCS, transcranial direct current stimulation; MBCT, mindfulness-based cognitive therapy.

Regarding the MADRS, no significant differences were found at t0 [*t*(29) = −0.641; *p* = 0.526 > 0.05] and t1 [*t*(29) = −1.149; *p* = 0.260] between the two treatment conditions, but a significant difference was found at t2 [*t*(29) = −2.854; *p* = 0.008**]. Newly generated pairwise comparisons showed significant differences across three times for the MBCT condition [between t0 and t1 (*p* = 0.001***), between t1 and t2 (*p* = 0.001***), and between t0 and t2 (*p* = 0.004**)], but for the relaxation condition, only between t0 and t1 (*p* = 0.001***) and between t0 and t2 (*p* = 0.009**). Indeed, no significant difference was found between t1 and t2 (*p* = 0.321 > 0.05) for the relaxation condition. Those results seem to indicate a significant improvement of depression across three times for the MBCT condition [t0 (*M* = 23.13; SD = 1.75); t1 (*M* = 13.73; SD = 2.47); t2 (*M* = 9.8; SD = 2.56)], but not for the relaxation condition, whose subjects seem to show a significant improvement between t0 (*M* = 24.75; SD = 1.8) and t1 (*M* = 17.25; SD = 1.84), but not between t1 and t2 (*M* = 19.06; SD = 2.02) (**Figure 2**).

Regarding the SARI, no significant differences were found at t0 [*t*(29) = −1.576; *p* = 0.126 > 0.05] and t1 [*t*(29) = −1.597; *p* = 0.121 > 0.05] between the two treatment conditions, but a significant difference was found at t2 [*t*(29) = −2.692; *p* = 0.012*]. Newly generated pairwise comparisons showed significant differences between t0 and t1 (*p* = 0.018**) and between t0 and t2 (*p* = 0.007**) and not between t1 and t2 (*p* = 1.000) for the MBCT condition, but only between t0 and t1 (*p* = 0.002**), and not between t1 and t2 (*p* = 0.097 > 0.05) nor between t0 and t2 (*p* = 0.924 > 0.05), for the relaxation condition. Those results seem to indicate a significant improvement of auto-evaluation of sadness ruminative behavior (SARI) at t1, and a stabilization of this score at t2 for the MBCT condition [t0 (*M* = 40.53; SD = 7.03); t1 (*M* = 34.67; SD = 10.16); t2 (*M* = 33.53; SD = 10.82)], but not for the relaxation condition, whose subjects seem to show a significant improvement between t0 (*M* = 45; SD = 8.6) and t1 (*M* = 40.5; SD = 10.16), but not between t1 and t2 (*M* = 43,69; SD = 10.17).

#### Cognitive Battery

A time effect was found between t0, t1, and t2 regarding all cognitive scores, that is, MOCA score [*F*(1,907, 55,296) = 24.575; *p* = 0.001***; *η*2 = 0.459; *p* = 1.000], the TMT score [*F*(2, 58) = 6.632; *p* = 0.003**; *η*2 = 0.186; *p* = 0.898], the Digit Span-F score [*F*(2, 58) = 12.326; *p* = 0.001***; *η*2 = 0.298; *p* = 0.994], and the Digit Span-B score [*F*(1,98, 57,426) = 7.513; *p* = 0.001***; *η*2 = 0.206; *p* = 0.931].

Importantly, no interaction effect between our within-subjects and between-subjects variables (time * treatment conditions) was observed for the MOCA score [*F*(1,907, 55,296) = 1.944; *p* = 0.155 > 0.05; *p* = 0.387], neither for the TMT score [*F*(2, 58) = 0.463; *p* = 0.632 > 0.05; *p* = 0.122], while an interaction effect between our within-subjects and between-subjects variables (time * treatment conditions) has been found regarding the Digit Span-F score [*F*(2, 58) = 9.259; *p* = 0.001***; *η*2 = 0.242; *p* = 0.971] and Digit Span-B score [*F*(1,98, 57,426) = 5.903; *p* = 0.005**; *η*2 = 0.169; *p* = 0.857].

Interestingly, when looking at automatically generated pairwise comparisons for the cognitive evolution at the MOCA, we observe that this score is significantly different between t0 and t1 (*p* = 0.001***), significantly different between t1 and t2 (*p* = 0.005**), and significantly different between t0 and t2 (*p* = 0.001***), indicating a significant improvement of general cognitive functioning (MOCA) at t1 and at t2 for both treatment conditions. When looking at automatically generated pairwise comparison for the cognitive evolution at the TMT, we observe that this score is significantly different between t0 and t1 (*p* = 0.05*); significantly different between t0 and t2 (*p* = 0.018**), and not significantly different between t1 and t2 (*p* = 1.000 > 0.05), indicating a significant improvement of mental flexibility (TMT) at t1 and a stabilization of those scores at t2 in both treatment conditions.

With the idea of exploring the observed interaction effect (time * treatment conditions) for the Digit Span-F and Digit Span-B scores, we conducted specific post-hoc analyses. After splitting our general sample into two independent samples, we conducted an independent *t*-test.

Regarding the Digit Span-F, no significant differences were found at t0 [*t*(29) = 1.023; *p* = 0.315 > 0.05] between the two treatment conditions, but a significant difference was found at t1 [*t*(29) = 2.146; *p* = 0.040*] and t2 [*t*(29) = 3.652; *p* = 0.001***] between the two treatment conditions. When looking at newly generated pairwise comparisons, we found significant differences across three times for the MBCT condition [between t0 and t1 (*p* = 0.042*), between t1 and t2 (*p* = 0.006**), and between t0 and t2 (*p* = 0.001***)] but no significant difference for the relaxation condition [between t0 and t1 (*p* = 0.505 > 0.05); between t0 and t2 (*p* = 1.000 > 0.05); between t1 and t2 (*p* = 0.408 > 0.05)]. Those results seem to indicate a significant improvement of the amount of retained numbers across three times for the MBCT condition [t0 (*M* = 8,06; SD = 0.49); t1 (*M* = 9,4; SD = 0.567); t2 (*M* = 10.33; SD = 0.558)], but not for the relaxation condition, whose subjects seem to show no significant improvement between t0 (*M* = 7,43; SD = 0.37), t1 (*M* = 7,87; SD = 0.437), and t2 (*M* = 7,56; SD = 0.516).

Regarding the Digit Span-B, no significant differences were found at t0 [*t*(29) = 0.551; *p* = 0.586 > 0.05] nor at t1 [*t*(29) = 1.443; *p* = 0.160] between the two treatment conditions, but a significant difference was found at t2 [*t*(29) = 3.242; *p* = 0.003**]. Further pairwise comparisons revealed a significant difference between t0 and t2 (*p* = 0.005**), but no significant difference between other times [between t1 and t2 (*p* = 0.097 > 0.05)], nor between t0 and t1 (*p* = 0.269) for the MBCT condition. For the relaxation condition, no significant difference has been found [between t0 and t1 (*p* = 1.000 > 0.05); between t0 and t2 (*p* = 1.000 > 0.05); between t1 and t2 (*p* = 1.000 > 0.05)]. Those results seem to indicate a significant improvement of working memory between t0 and t2 for the MBCT condition [t0 (*M* = 5.6; SD = 1.95); t1 (*M* = 6.40; SD = 2.23); t2 (*M* = 7.53; SD = 2.13)], but not for the relaxation condition, whose subjects seem to show no significant improvement between t0 (*M* = 5,25; SD = 1.57), t1 (*M* = 5,38; SD = 1.7), and t2 (*M* = 5,38; SD = 1.54).

No correlations were found between clinical and cognitive variables.

## Discussion

### Obtained Results

Our previously described results seem to indicate a positive impact of both combined treatment conditions on the clinical and cognitive symptoms of depression assessed by MADRS, SARI, STAI, MOCA, Digit Span-F, Digit Span-B, and TMT at t1. However, a treatment condition effect has been found at t2 regarding depression (MADRS), sadness ruminative behavior (SARI), the number of retained numbers (Digit Span-F), and working memory (Digit Span-B). Those results seem to indicate a sustained efficacy at t2 of the tDCS–MBCT condition compared with the tDCS relaxation. We stipulated that tDCS and MBCT might act complementarily with MBCT, allowing to avoid relapse after the end of tDCS sessions. We, therefore, expected an improvement of cognitive deficits in depressed patients, a diminution of ruminations, and lower depression scores after tDCS treatment to be better maintained through time in the MBCT condition compared with the relaxation condition.

As a matter of fact, patients included in the relaxation condition (active tDCS on the left PFDLC + relaxation) display an improvement of depression (MADRS), sadness ruminative behavior (SARI), anxiety (STAI), working memory (Digit Span B), general cognitive functioning (MOCA), and mental flexibility (TMT) at t1. However, most of those scores stay stable at t2 (MADRS, STAI, SARI, MOCA, TMT, and Digit Span-F), indicating no significant improvement between t1 and t2 for the participants of the relaxation condition. As relaxation is being considered as a control condition for MBCT and other psychotherapeutic modalities such as CBT and/or interpersonal therapy (IPT) (49, 50) in depression, it might be stipulated that the positive effect observed in patients who received the relaxation condition is due to the tDCS’s impact. Indeed, in 2008, a review of 15 trials exploring the possibility of using relaxation as a first-line treatment for depression confirmed that relaxation was slightly better than no treatment or minimal treatment but not as effective as psychological therapies like CBT (59).

As the only subjects who seem to show a further significant improvement of depression (MADRS), auto-assessment of sadness ruminative behavior (SARI), the number of retained numbers (Digit Span-F), and working memory (Digit Span-B) between t1 and t2 are patients who received the MBCT condition, it might be suggested that combining tDCS and MBCT may be of clinical relevance for a longer-term maintenance of clinical improvements. This better efficacy of the MBCT condition for the maintenance of clinical improvements might be linked to the combined use of tDCS and MBCT. Both of those therapeutic techniques are influencing the same neural circuits and boosting related cognitive deficits such as attention, working memory, and executive functioning, thereby allowing patients to disengage from ruminations and allowing them to improve their clinical state.

We tested a new therapeutic setting combining tDCS with MBCT in patients with drug-resistant depression. Obtained results seem to support our hypothesis stipulating that tDCS and MBCT might have complementary action mechanisms allowing patients to disengage from ruminations, which is considered as a main causal and maintenance factor of depression. However, some methodological considerations might have impacted our results. We present those limitations below with the idea of proposing new research parameters. Clinical implications are further discussed.

### Limitations and Perspectives

If obtained results are encouraging for the treatment of patients with resistant depression and that this study was of clinical relevance, we should to point out the difficulties we had to recruit and to carefully select therapy-resistant depressed patients. For instance, some depressed patients dropped out of our protocol, invoking some reasons such as their work schedules and mainly to the highly energy-demanding protocol. Indeed, one of the classical symptoms of depression is the lack of energy and motivation to get things done. Our research protocol was inevitably a highly demanding program in terms of time and energy for patients, as well as for the therapeutic team in charge of both tDCS and MBCT. That is, the sample sizes presented in our pilot study are smaller than the estimated number of patients we would need for further randomized controlled trial (RCT) study and this small sample size limits analyses of other variables such as the impact the type of antidepressant treatment [selective serotonin reuptake inhibitor (SSRI), serotonin and norepinephrine reuptake inhibitor (SNRI), and SSRI + SNRI] and of comorbidities with other psychiatric conditions such as anxious disorder [obsessive compulsive disorder (OCD) and social phobia]. But other considerations should also been highlighted.

First, about the tDCS use, this study explores the efficacy of a new combined-treatment model that includes a recent neuromodulation technique, as encouraging results have been found regarding the clinical outcomes of tDCS’s applications in patients suffering from depression. However, it also appears that no clear guidelines yet exist considering this tDCS technique, as the uncertainty principally remains regarding some technical and neuroanatomical considerations, such as the electrodes montages (two main montages exist for the application of tDCS in depression: active anodal tDCS of the left DLPFC, and the cathode is placed either over the right DLPFC or over the right orbitofrontal cortex), the current intensity, and the session repetition timing. That is, according to the very recent literature review of Lefaucheur et al. (22), numerous factors are thought to modulate the impact of tDCS on cortical excitability [distance and orientation of the axonal somatodendritic axis with respect to the electric field; baseline activity of neural networks and afferent synaptic inputs to direct current; spatial relationships between the stimulated active target field, its projections areas, and the resting surrounding structures; pathological alterations of transmitter systems; medication taken by patients (30); individual polymorphisms and the current intensity (60); session repetition timing (61, 62); and electrode sizes, areas, shapes, or montage]. Therefore, clinical effects provided by tDCS may vary across patients and studies according to many technical and neuroanatomical considerations and relationships (22). When designing protocols and interpreting the impacts of tDCS in patients with depression and other neuropsychiatric diseases, all these factors should definitely be taken into account. In our study, electrode sizes (35 cm2), shapes (rectangular), and montage have consistently been controlled. But some other factors might have influenced our patients’ stimulations such as the baseline activity of neural networks and individual polymorphisms.

Also, in our study, we used a current intensity of 2 mA. Nevertheless, a recent interesting communication of Vöröslakos et al. (63) establish that neuronal circuits are affected by intensity currents that are higher than those used in conventional protocols such as 2 mA. Further research definitely should continue to explore the adequate tDCS parameters in terms of current intensity, electrodes montages, and stimulation duration.

Importantly, some recent studies (64) have found that significant reduction in depressive symptoms with tDCS appears after 6 weeks of treatment, while our protocol was designed for a first block of treatment of eight consecutive days and a second block of treatment including a single remind session 2 weeks after the first treatment block and display a short-time follow-up. The short-time treatment and follow-up of our protocol could obviously have impacted obtained results. The next research should take into account this point and should assess if a longer duration of our treatment model combining tDCS with MBCT might be even more efficient in order to reduce depression symptoms.

Second, about studying a sample of patients with resistant depression, some, such as Bennabi et al. (65), have suggested that tDCS efficacy diminishes in patients with high therapeutic resistance. Brunoni et al. (66) confirmed this observation in his study by showing that tDCS efficacy seems to be negatively correlated with the degree of treatment resistance. Not only could this observation have had an impact on our obtained results, but it would also be of major interest to explore the efficacy of our combined treatment model on a population of patients with a first depressive episode and non-drug-resistant depression.

Third, regarding an MBCT consideration, it is important to note that the MBCT program used in this study is normally delivered in eight weekly 2-h group training sessions. In our study, we have used a modified version of the classical program in order to directly combine the eight classical MBCT sessions with eight close tDCSs, that is, daily 2-h group training sessions. It might have affected the way patients have benefited of the MBCT tools, as the time accorded for their learning was shorter than initially defined. However, it has been proposed that most mindfulness-based interventions utilizing some forms of meditation practice result in objective cognitive and affective changes relative to the subtle and subjective shifts in awareness (67).

Also, the classical program proposed by Segal et al. (34) has initially been conceived as a program to prevent depressive relapses in stabilized patients, and not for acute depressed patients (34, 68), mostly because different cognitive, affective, and motivational characteristics of acute depression have been proposed to hinder the process of this specific 8-week therapy (68, 69). In our study, included patients presented a baseline score of 23.13 (MBCT condition) and 24.75 (relaxation condition) at the MADRS. This observation may have impacted our results in the way the offered mindfulness program might not have been as effective as it should, considering the high depression scores of patients at baseline. However, other authors have showed that the MBCT program positively impacts depressive symptom severity (39).

Finally, the way we initially designed the protocol might have had an impact on obtained results. Indeed, the protocol design generates a lack of blinding of the investigator, who was the same person responsible of animating the two treatment groups. The way we combined tDCS and psychotherapeutic modalities might also have an impact. Indeed, our patients received their assigned psychotherapeutic modalities right after the tDCS. However, according to some authors, the time sequence between tDCS and psychotherapeutic practice could be a major issue, as synergic effects appear to be maximal when the active stimulation takes place during the psychotherapy rather than before it (70).

## Conclusion

The combined use of tDCS and mindfulness may be of interest in resistant depression patients: MBCT might provide a mechanism to keep the tDCS beneficial effects through time and even to improve them. Further research should definitely continue to investigate the adequate tDCS parameters and the efficacy of our combined treatment model in a population of patients with a first depressive episode and non-drug-resistant depression and also in a greater sample size and should explore if a synergic effect could be enhanced when the active stimulation takes place during the psychotherapy rather than before it.

## Ethics Statement

This study was carried out in accordance with the recommendations of “Comité Ethique Hospitalier OM026 du CHU Brugmann” and of “Comité d’Ethique ISPPC. OM800” with written informed consent from all subjects. All subjects gave written informed consent in accordance with the Declaration of Helsinki. The protocol was approved by the “Comité Ethique Hospitalier OM026 du CHU Brugmann” and of “Comité d’Ethique ISPPC. OM800”.

## Author Contributions

All authors listed, have made substantial, direct and intellectual contribution to the work, and approved it for publication.

## Funding

This research was supported by IRIS Funds and A.B. Funds.

## Conflict of Interest

The authors declare that the research was conducted in the absence of any commercial or financial relationships that could be construed as a potential conflict of interest.
